# Mechanical Stimulation-Induced Calcium Signaling by Piezo1 Channel Activation in Human Odontoblast Reduces Dentin Mineralization

**DOI:** 10.3389/fphys.2021.704518

**Published:** 2021-08-24

**Authors:** Mayumi Matsunaga, Maki Kimura, Takehito Ouchi, Takashi Nakamura, Sadao Ohyama, Masayuki Ando, Sachie Nomura, Toshifumi Azuma, Tatsuya Ichinohe, Yoshiyuki Shibukawa

**Affiliations:** ^1^Department of Physiology, Tokyo Dental College, Tokyo, Japan; ^2^Department of Dental Anesthesiology, Tokyo Dental College, Tokyo, Japan; ^3^Department of Biochemistry, Tokyo Dental College, Tokyo, Japan

**Keywords:** dentin, odontoblast, Piezo channel, mechanosensitive channel, tooth pain, orofacial pain

## Abstract

Odontoblasts play critical roles in dentin formation and sensory transduction following stimuli on the dentin surface. Exogenous stimuli to the dentin surface elicit dentinal sensitivity through the movement of fluids in dentinal tubules, resulting in cellular deformation. Recently, Piezo1 channels have been implicated in mechanosensitive processes, as well as Ca^2+^ signals in odontoblasts. However, in human odontoblasts, the cellular responses induced by mechanical stimulation, Piezo1 channel expression, and its pharmacological properties remain unclear. In the present study, we examined functional expression of the Piezo1 channel by recording direct mechanical stimulation-induced Ca^2+^ signaling in dentin matrix protein 1 (DMP-1)-, nestin-, and dentin sialophosphoprotein (DSPP)-immunopositive human odontoblasts. Mechanical stimulation of human odontoblasts transiently increased intracellular free calcium concentration ([Ca^2+^]_i_). Application of repeated mechanical stimulation to human odontoblasts resulted in repeated transient [Ca^2+^]_i_ increases, but did not show any desensitizing effects on [Ca^2+^]_i_ increases. We also observed a transient [Ca^2+^]_i_ increase in the neighboring odontoblasts to the stimulated cells during mechanical stimulation, showing a decrease in [Ca^2+^]_i_ with an increasing distance from the mechanically stimulated cells. Application of Yoda1 transiently increased [Ca^2+^]_i_. This increase was inhibited by application of Gd^3+^ and Dooku1, respectively. Mechanical stimulation-induced [Ca^2+^]_i_ increase was also inhibited by application of Gd^3+^ or Dooku1. When Piezo1 channels in human odontoblasts were knocked down by gene silencing with short hairpin RNA (shRNA), mechanical stimulation-induced [Ca^2+^]_i_ responses were almost completely abolished. Piezo1 channel knockdown attenuated the number of Piezo1-immunopositive cells in the immunofluorescence analysis, while no effects were observed in Piezo2-immunopositive cells. Alizarin red staining distinctly showed that pharmacological activation of Piezo1 channels by Yoda1 significantly suppressed mineralization, and shRNA-mediated knockdown of Piezo1 also significantly enhanced mineralization. These results suggest that mechanical stimulation predominantly activates intracellular Ca^2+^ signaling *via* Piezo1 channel opening, rather than Piezo2 channels, and the Ca^2+^ signal establishes intercellular odontoblast-odontoblast communication. In addition, Piezo1 channel activation participates in the reduction of dentinogenesis. Thus, the intracellular Ca^2+^ signaling pathway mediated by Piezo1 channels could contribute to cellular function in human odontoblasts in two ways: (1) generating dentinal sensitivity and (2) suppressing physiological/reactional dentinogenesis, following cellular deformation induced by hydrodynamic forces inside dentinal tubules.

## Introduction

Piezo proteins are large membrane proteins and components of mechanosensitive non-selective cationic channels (Bagriantsev et al., 2014; Beech and Xiao, 2018). Piezo channels are classified into two subtypes: Piezo1 (encoded by the *FAM38A* gene) and Piezo2 (encoded by the *FAM38B* gene; Bagriantsev et al., 2014; Morozumi et al., 2020). In mammals, Piezo1 is mainly expressed in cells with essential mechanosensory functions, such as skin, bladder, kidney, lung, endothelial cells, erythrocytes, and periodontal ligament cells (Bagriantsev et al., 2014; Borbiro and Rohacs, 2017). Piezo1 is required for vascular development, stretch-induced epithelial proliferation, crowdsensing, urinary osmolarity regulation, and neuronal stem cell lineage choice (Beech and Xiao, 2018; Dalghi et al., 2019). In contrast, Piezo2 is prominently expressed in the lung, bladder, dorsal root ganglia, and trigeminal ganglia (Coste et al., 2010; Bagriantsev et al., 2014). Piezo2 is also required for the sensation of touch in the Merkel cells of the skin, proprioception, airway stretch, and respiration (Woo et al., 2014; Chesler et al., 2016; Nonomura et al., 2017).

Odontoblasts have been reported to express Piezo1 and Piezo2 channels (Khatibi Shahidi et al., 2015; Miyazaki et al., 2019). In addition, Piezo1 channels in rat odontoblasts play an essential role in mechanosensory processes as mechanosensitive ion channels and mediate the intracellular Ca^2+^ signaling pathway to release neurotransmitters, establishing neural communication between odontoblasts and the intradental Aδ neurons (Sato et al., 2018). Odontoblast-neuron communication generates action potential on the neurons, resulting in the development of dentinal sensitivity (odontoblast hydrodynamic receptor model; Shibukawa et al., 2015; Sato et al., 2018). However, the detailed functional properties of Piezo1 channel in odontoblasts remain to be clarified. To further investigate the functional expression and pharmacological profiles of Piezo channels and their cellular functions, we examined mechanical stimulation- and ligand-evoked intracellular Ca^2+^ signaling by Piezo channel activation in human odontoblasts. We also investigated the effects of Piezo1 channel knockdown by gene silencing with short hairpin RNA (shRNA) on the direct mechanical stimulation-induced intracellular free Ca^2+^ concentration ([Ca^2+^]_i_) increases. In addition, we examined the role of Piezo1 channels during dentinogenesis using mineralizing assays.

## Materials and Methods

### Cell Culture

**Figure 1:**
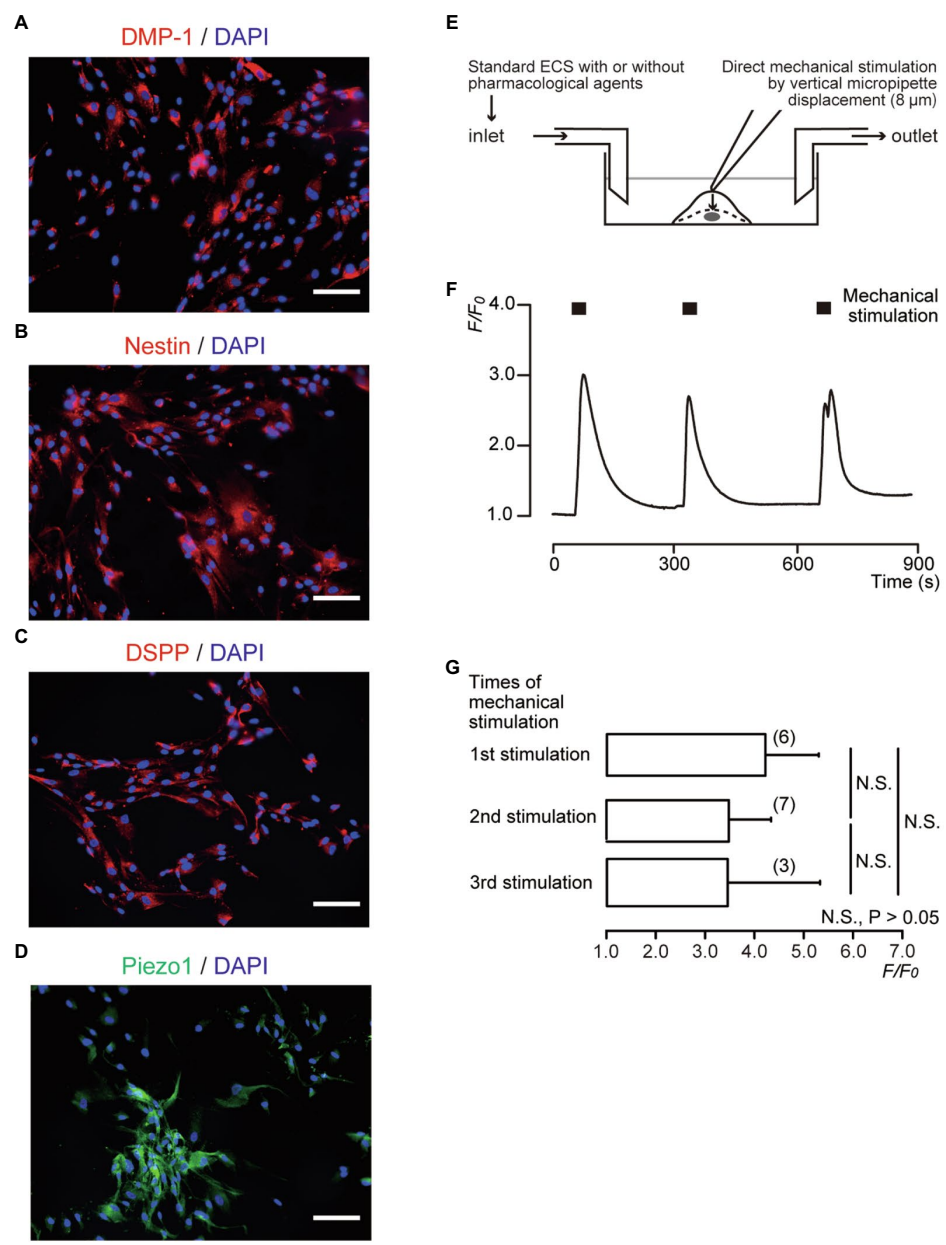
Direct mechanical stimulation transiently increased [Ca^2+^]_i_ in dentin matrix protein 1 (DMP-1)-, nestin-, and dentin sialophosphoprotein (DSPP)-immunopositive human odontoblasts. **(A–D)** Human odontoblasts showed positive immunoreactivity to the DMP-1 (red in **A**), nestin (red in **B**), DSPP (red in **C**), and Piezo1 channels (green in **D**). Nuclei were represented in blue. Scale bar: 100μm. No fluorescence was detected in the negative controls (not shown). **(E)** Experimental setup for the measurement of Ca^2+^-sensitive dye fluorescence. Standard extracellular solution (standard ECS) with or without pharmacological Piezo1 channel activator/inhibitors was applied from inlet by a rapid gravity-fed perfusion system and aspirated using a vacuum pump. Diagram also shows mechanical stimulation induced by vertical micropipette displacement downward by 8.0μm. **(F)** Representative trace of transient increases in [Ca^2+^]_i_ during repeated mechanical stimulations (black boxes). **(G)** Bar graph showing the *F*/*F*_0_ values as a function of numbers of mechanical stimuli by vertical micropipette displacement (8μm). Each bar indicates the mean±SD. Numbers in parentheses indicate the number of experimented cells. N.S., not significant.

Human odontoblast cells were obtained from a healthy third molar and immortalized by transfection with the human telomerase transcriptase gene (Kitagawa et al., 2007; Ichikawa et al., 2012; Kimura et al., 2016; Kojima et al., 2017). The resulting cells showed mRNA expression of dentin sialophosphoprotein (DSPP), type 1 collagen, alkaline phosphatase, and bone sialoprotein, and exhibited nodule formation by Alizarin red staining in the mineralizing medium (Kitagawa et al., 2007). Note that these cells were immunopositive for odontoblast marker proteins (Figure 1). The cells were gifted by Dr. Takashi Muramatsu of Tokyo Dental College, Japan. Human odontoblasts were cultured in basal medium [alpha-minimum essential medium containing 10% FBS, 1% penicillin–streptomycin (Life Technologies, Tokyo, Japan), and amphotericin B (Sigma-Aldrich, St. Louis, MO, United States)] at 37°C in a humidified atmosphere of 5% CO_2_ for 48h. The odontoblast suspension was adjusted to a density of 5×10^4^ cells/ml.

### Solutions and Reagents

Standard extracellular solution (standard ECS) containing (in mM) 136 NaCl, 5 KCl, 2.5 CaCl_2_, 0.5 MgCl_2_, 10 HEPES, 10 glucose, and 12 NaHCO_3_ (adjusted to pH 7.4 with Tris) was used as the extracellular solution. A selective pharmacological Piezo1 channel activator, Yoda1 (Syeda et al., 2015), was obtained from Cayman Chemical Company (Ann Arbor, MI, United States). A non-selective mechanosensitive ion channel inhibitor, GdCl_3_ (Gd^3+^; Ermakov et al., 2010), and a pharmacological competitive antagonist of Yoda1, Dooku1 (Evans et al., 2018), were obtained from R&D Systems, Inc. (Minneapolis, MN, United States). Stock solutions of Yoda1 and Dooku1 were prepared in dimethyl sulfoxide. The other was prepared using ultrapure water (Millipore, MA, United States). Stock solutions were diluted with standard ECS to an appropriate concentration before use.

### Direct Mechanical Stimulation of Single Human Odontoblast

Direct mechanical stimulation (Sato et al., 2015; Shibukawa et al., 2015; Nishiyama et al., 2016) was applied using a fire-polished glass micropipette with a tip diameter of 2–3μm, which was filled with standard ECS. Micropipettes were pulled from capillary tubes using a DMZ Universal Puller (Zeitz Instruments, Martinsried, Germany). The micropipette was operated using a micromanipulator (UMp Micromanipulators; Sensapex, Oulu, Finland). The tip was positioned just above the target human odontoblast membrane, and the micropipette was moved vertically downward by 8.0μm at a velocity of 2.0μm/s (Sato et al., 2015; Shibukawa et al., 2015) to generate a focal mechanical stimulation (Figure 1E). Stimulation was applied for 22s, after which the pipette was retracted at the same velocity. The stimulations were applied in series no more than three times to avoid unfavorable cell damage.

### Measurement of Ca^2+^-Sensitive Dye Fluorescence

Human odontoblasts were incubated for 60min at 37°C and 5% CO_2_ in a standard solution containing 10μM fura-2-acetoxymethyl ester (Dojindo Laboratories, Kumamoto, Japan) and 0.1% (*w*/*v*) pluronic acid F-127 (Life Technologies). They were then washed with a fresh standard solution. A dish containing fura-2-loaded human odontoblasts was mounted on the stage of a microscope (IX73; Olympus, Tokyo, Japan), which was installed with an HCImage system (Hamamatsu Photonic, Shizuoka, Japan), an excitation wavelength selector, and an intensified charge-coupled device camera system. Fura-2 fluorescence emission was recorded at 510nm in response to alternating excitation wavelengths of 340nm (F340) and 380nm (F380). The intracellular free Ca^2+^ concentration ([Ca^2+^]_i_) was defined as the fluorescence ratio (*R*_F340/F380_) at two excitation wavelengths and then described as *F*/*F*_0_ units; the *R*_F340/F380_ value (*F*) was normalized to the resting value (*F*_0_). All the experiments were performed at room temperature (28°C). Standard ECS with or without pharmacological Piezo1 channel activator/inhibitors was applied by superfusion using a rapid gravity-fed perfusion system (ValveLink8.2 Controller; AutoMate Scientific, Berkeley, CA, United States; Figure 1E).

### Measurement of Intercellular Distance

Human odontoblasts were imaged using an intensified charge-coupled device camera (Hamamatsu Photonic) and microscope (Olympus). The distance from a mechanically stimulated human odontoblast to each neighboring cell was determined from the images obtained (HCImage) by measuring the shortest distance between each pair of cells.

### Piezo1 Channel Knockdown by Gene Silencing With Short Hairpin RNA

To generate the odontoblasts transfected by shRNA, including vectors specific for human Piezo1 (shRNA-Piezo1 transfected cells) or an empty vector control (shRNA-Control transfected cells), the Lenti-X™ 293T cell line (Takara Bio Inc., Shiga, Japan) was used to produce lentiviral vector particles. Packaging plasmids pCAG-HIVgp (RIKEN BioResource Research Center, Tsukuba, Japan, RDB04394) and pCMV-VSV-G-RSV-Rev (RIKEN BioResource Research Center, RDB04393) were used for lentiviral vector packaging. Lentiviral vectors specific for human Piezo1 (Sigma-Aldrich) and a TRC2 empty vector control (Sigma-Aldrich) were used in this experiment. Viral supernatants were harvested after 72h. Human odontoblasts with 60–80% confluency were transfected with lentiviral vector particles including shRNA and then incubated under normal growth conditions for 72h.

### Immunostaining

Human odontoblasts were transferred to eight-well glass chambers (AGC Techno Glass Co., Ltd., Shizuoka, Japan) and maintained under culture conditions as described above. Cells were fixed with 4% paraformaldehyde (FUJIFILM Wako Pure Chemical Co., Osaka, Japan) and washed with 1× PBS (Thermo Fisher Scientific, Tokyo, Japan). After 10min of incubation with blocking buffer (Nacalai Tesque, Kyoto, Japan) at room temperature, the following primary antibodies were applied for 6h: mouse monoclonal anti-dentin matrix protein 1 (DMP-1; 1:200; sc-73,633, LFMb-31; Santa Cruz Biotechnology, Dallas, Texas, United States), mouse monoclonal anti-DSPP (1:200; sc-73,632, LFMb-21; Santa Cruz Biotechnology), or mouse monoclonal anti-nestin (1:200; sc-23927, 10c2; Santa Cruz Biotechnology). The secondary antibody (Alexa Fluor® 555 donkey anti-mouse; Thermo Fisher Scientific) was then applied for 1h. Human odontoblasts, which were transfected with shRNA-Piezo1 and shRNA-Control, were also transferred to eight-well glass chambers (AGC Techno Glass Co., Ltd.), maintained in culture conditions, and fixed with 4% paraformaldehyde (FUJIFILM Wako Pure Chemical Co.). The cells were washed with 1× PBS (Thermo Fisher Scientific K.K.), and incubated for 10min with blocking buffer (Nacalai Tesque) at room temperature. Next, Alexa Fluor® 488 conjugated rabbit polyclonal anti-Piezo1 (1:200; NBP1-78537 AF488; Novus Biologicals, LLC, Centennial, CO, United States) and Alexa Fluor® 594 conjugated rabbit polyclonal anti-Piezo2 (1:200; NBP1-78538 AF594; Novus Biologicals) were applied for 6h. Stained samples were mounted in mounting medium with 4,6-diamidino-2-phenylindole (Abcam, Cambridge, United Kingdom). Immunostaining was observed and analyzed, and photographs were taken (as 8-bit TIFF images) using a fluorescence microscope (BZ-X710; Keyence, Osaka, Japan). For the negative control, the cells were incubated with non-immune antibodies diluted to equivalent concentrations to those of the primary antibodies (data not shown). For shRNA-Piezo1 transfected and shRNA-Control transfected cells, TIFF images obtained with 8-bit of 1,920×1,440 pixels were converted to reversed grayscale. Then, the percentage area of the Piezo1- or Piezo2-immunopositive cells was measured using automated thresholds of ImageJ software (NIH, Bethesda, MD, United States).

### Mineralization Assay

Human odontoblasts were grown to full confluency in basal medium, and then transferred to mineralization medium (10mM β-glycerophosphate and 100μg/ml ascorbic acid in basal medium) for growth at 37°C in 5% CO_2_. To determine the effects of Piezo1 activity on mineralization, the cells were cultured in mineralization medium without (as control) or with the pharmacological Piezo1 modifier, Yoda1 (2μM), Dooku1 (10μM), or Yoda1 (2μM) and Dooku1 (10μM) for 28days. The odontoblasts transfected with shRNA, including vectors specific for human Piezo1, or including an empty vector control (see above), were also grown to full confluency in basal medium, and then transferred to mineralization medium at 37°C in 5% CO_2_ for 28days. During the 28-day culture period, mineralization media with or without pharmacological agents were changed twice a week (Kimura et al., 2016). To detect calcium deposition, the cells were subjected to Alizarin red staining, and the mineralizing efficiencies were measured using ImageJ software (NIH). Images were obtained with a digital camera (Sony, Tokyo, Japan), converted to 32-bit, and converted into reversed grayscale. Regions of interest (ROIs) were then determined for each whole well to measure the mean luminance intensities of the total pixel numbers (I) of the ROI. Mineralizing efficiencies were normalized and represented as *I*/*I*_0_ units, and the intensities (*I*) of Alizarin red staining were normalized to the mean intensity values of areas without cells (*I*_0_).

### Statistical Analysis

Data are expressed as the mean±SE or SD of the mean of *N* observations, where *N* represents the number of experiments or cells tested. The Kruskal-Wallis test, Friedman test, and Dunn’s *post-hoc* test were used to determine non-parametric statistical significance. Parametric statistical significance was determined using a two-tailed Student’s *t*-test to analyze the percentage areas of Piezo1/2 channel-immunopositive cells in the immunofluorescence analyses. Statistical significance was set at *p*<0.05. Statistical analysis was performed using GraphPad Prism 5.0 (GraphPad Software, La Jolla, CA, United States).

## Results

### Direct Mechanical Stimulation to Human Odontoblasts Transiently Increased [Ca^2+^]_i_

We observed distinct DMP-1, nestin, and DSPP immunoreactivity in human odontoblasts (Figures 1A–C). In addition, odontoblasts also expressed Piezo1 immunoreactivity (Figure 1D). Cells expressing odontoblast marker proteins were loaded with fura-2, and [Ca^2+^]_i_ was measured. When we applied mechanical stimulations to single human odontoblasts using a glass micropipette moving vertically (8μm) in a downward direction from a position just above the surface (0μm; Figure 1E), in the presence of extracellular Ca^2+^, [Ca^2+^]_i_ was increased to a peak value of 4.20±1.08*F*/*F*_0_ units for first stimulation (*N*=6; Figure 1F). After [Ca^2+^]_i_ returned to near-resting levels, further application of mechanical stimulation transiently increased [Ca^2+^]_i_ in human odontoblasts. Repeated mechanical stimulation did not show any desensitizing effect on the [Ca^2+^]_i_ increases after the third application (*p*>0.05; Figures 1F,G).

### Direct Mechanical Stimulation to Human Odontoblasts Transiently Increased [Ca^2+^]_i_ in Stimulated Human Odontoblasts and Neighboring Cells to the Stimulated Cells

**Figure 2:**
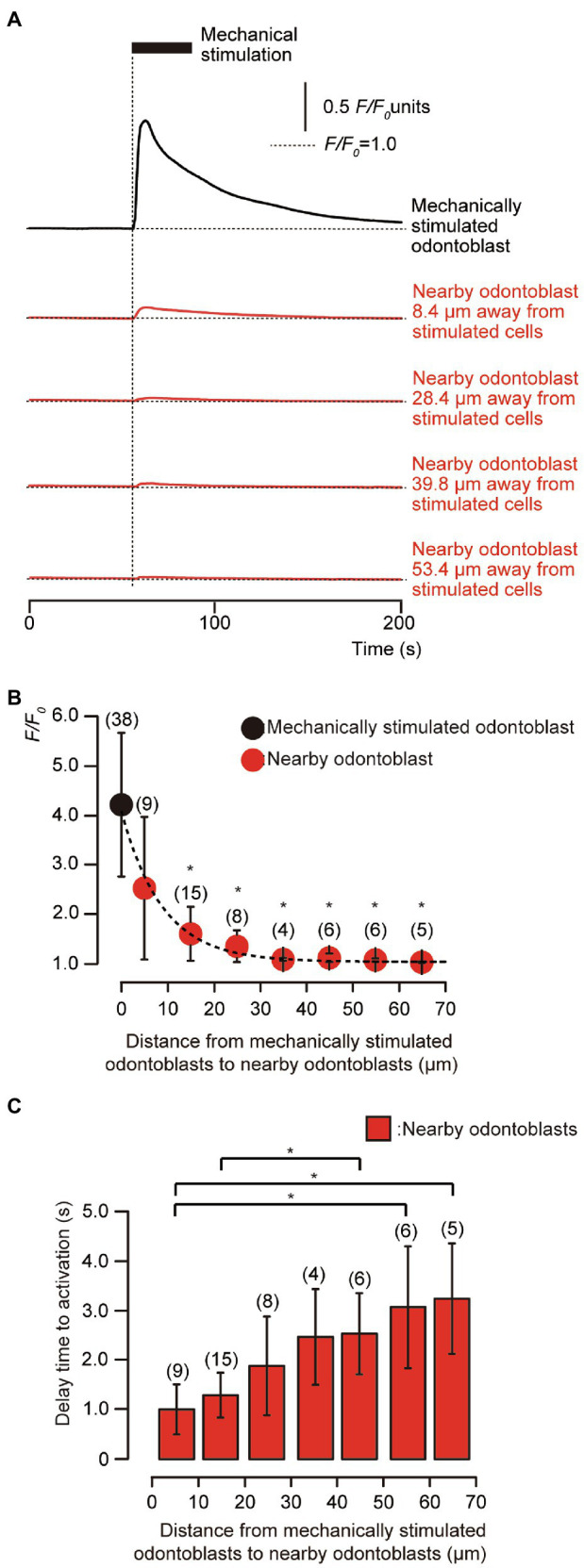
Transient [Ca^2+^]_i_ increases in mechanical-stimulated human odontoblast and neighboring human odontoblasts to the stimulated cells. **(A)** Representative traces indicating transiently increased [Ca^2+^]_i_ from a mechanically stimulated human odontoblast (black line) and neighboring human odontoblasts (red lines) in standard extracellular solution. Horizontal dotted lines indicate the baseline (*F*/*F*_0_=1.0) for each response, while vertical dotted line represents the time when mechanical stimulation was applied. The black box at the top shows the timing of mechanical stimulation by the displacement of a micropipette to a depth of 8.0μm. Responses from the nearby human odontoblasts were recorded in cells at 8.4μm, 28.4μm, 39.8μm, and 53.4μm away from the stimulated human odontoblast. **(B)** The *F*/*F*_0_ values of the mechanically stimulated odontoblasts (black circle) and nearby odontoblasts (red circles) located within 0–10μm, 11–20μm, 21–30μm, 31–40μm, 41–50μm, 51–60μm, and 61–70μm from the stimulated human odontoblasts are shown. The kinetics of the distance-dependent decrease of [Ca^2+^]_i_ were described well by the single exponential function (dotted line), showing spatial constants of the distance-dependent decrease of 6.11±0.76μm. Circles indicate the mean±SD. Numbers in parentheses indicate the number of cells tested. The [Ca^2+^]_i_ increases in neighboring odontoblasts were reduced by increasing their distance from mechanically stimulated cells. **(C)** Bar graph representing the mean time to increases in [Ca^2+^]_i_ in neighboring odontoblasts, during the mechanical stimulation to single odontoblasts. The delay time in [Ca^2+^]_i_ increase was measured by calculating the time between the onset of the mechanical stimulation of the odontoblasts and time require to achieve the rising phase for each [Ca^2+^]_i_ response in neighboring cells. Each data point indicates the mean±SD from the number of cells tested (numbers in parentheses). Statistically significant differences in **(B,C)** between values are indicated by asterisks. ^*^p<0.05.

In the presence of extracellular Ca^2+^, during the application of focal and direct mechanical stimulation to single human odontoblasts (8.0μm in-depth), we observed transient increases in [Ca^2+^]_i_ in the mechanically stimulated human odontoblasts (black line, Figure 2A) to a peak value of 4.21±1.46 *F*/*F*_0_ units (*N*=38; black circle, Figure 2B). In addition, we observed transient increases in [Ca^2+^]_i_ in the neighboring human odontoblasts (red lines, Figure 2A) during mechanical stimulation. Note that these cells were not in physical contact with each other. The plots in Figure 2B show *F*/*F*_0_ values as a function of the distance from the stimulated odontoblast (black circle) to neighboring odontoblasts (red circles). The peak values of [Ca^2+^]_i_ were 2.52±1.45 (*N*=9), 1.59±0.55 (*N*=15), 1.34±0.32 (*N*=8), 1.07±0.03 (*N*=4), 1.11±0.09 (*N*=6), 1.06±0.03 (*N*=6), and 1.02±0.01 (*N*=5) *F*/*F*_0_ units in human odontoblasts located within distances of 0–10μm, 11–20μm, 21–30μm, 31–40μm, 41–50μm, 51–60μm, and 61–70μm from mechanically stimulated human odontoblasts, respectively (red circles, Figure 2B). The amplitude of the [Ca^2+^]_i_ increases in neighboring human odontoblasts was reduced by increasing their distance from mechanically stimulated human odontoblasts. The kinetics of the distance-dependent attenuation of [Ca^2+^]_i_ were described well by a single exponential function (dotted line, Figure 2B). The spatial constants of the distance-dependent decrease in [Ca^2+^]_i_ were 6.11±0.76μm for neighboring odontoblasts. In addition, we observed a delay in the increase in [Ca^2+^]_i_ in the nearby odontoblasts during the mechanical stimulation of single odontoblasts (time when the mechanical stimulation applied to the stimulated odontoblasts was set to 0s; vertical dotted line, Figure 2A). The time delay increased with an increase in the distance from the mechanically stimulated odontoblasts (Figure 2C). The significant time delay in the increase in [Ca^2+^]_i_ in the neighboring odontoblasts located 51–60μm and 61–70μm away from mechanically stimulated odontoblasts was observed compared to that in the cells located at 0–10μm (Figure 2C). The time delay was ranged from 0.95±0.47s at 0–10μm away to 3.03±1.04s at 61–70μm away from mechanically stimulated cells.

### [Ca^2+^]_i_ Is Increased by a Selective Pharmacological Piezo1 Channel Activator

**Figure 3:**
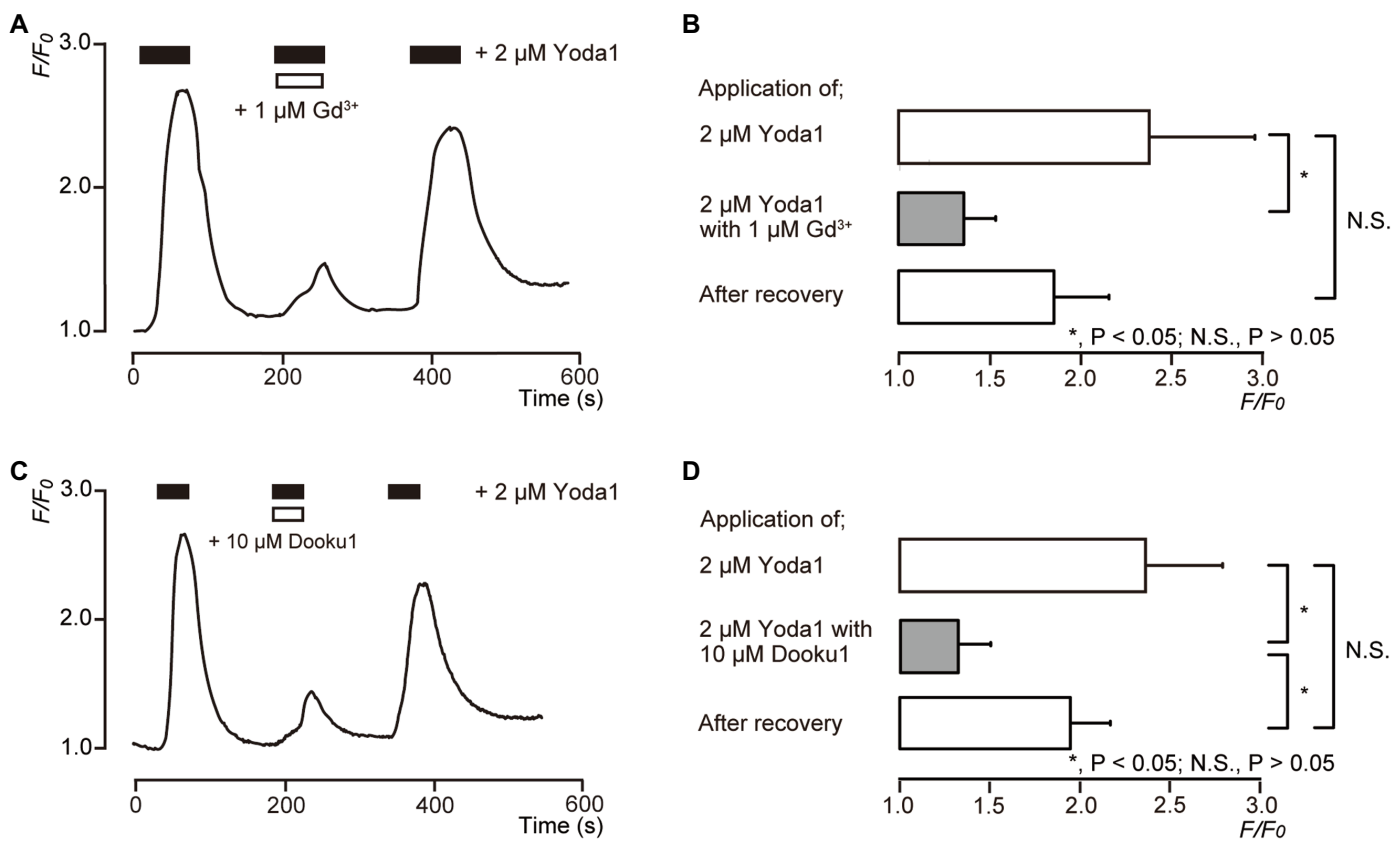
Piezo1 channel activator induces [Ca^2+^]_i_ increases in human odontoblasts. **(A,C)** Representative traces of transient [Ca^2+^]_i_ increase in response to 2μM Yoda1 in standard extracellular solution are shown. Addition of 1μM Gd^3+^ **(A)** or 10μM Dooku1 **(C)** inhibited Yoda1-induced [Ca^2+^]_i_ increases. In each figure, the boxes indicate the timings when the test solution, including pharmacological activator (black boxes at the top) or inhibitors (white boxes at the top), were applied. **(B,D)** Bar graphs representing values of *F*/*F*_0_ for the increases in [Ca^2+^]_i_ by 2μM Yoda1 with (gray columns) or without (open columns) 1μM Gd^3+^ **(B)** or 10μM Dooku1 **(D)**. The pharmacological activators and inhibitors used are indicated on the left side of the graphs. The resting value is defined as *F*/*F*_0_=1.0. Each bar indicates the mean±SE of six **(B)** and 12 **(D)** experiments. Asterisks denote statistically significant differences between columns (shown by solid lines): ^*^p<0.05.

To pharmacologically activate Piezo1 channels in human odontoblasts, Yoda1 was used as a selective Piezo1 channel activator. In the presence of external Ca^2+^ (2.5mM), the application of 2μM Yoda1 to human odontoblasts showed a rapid and transient [Ca^2+^]_i_ increase (Figures 3A,C), reaching a peak value of 2.38±0.58 (*N*=6; Figure 3B), and 2.36±0.43 (*N*=12) in *F*/*F*_0_ units (Figure 3D), followed by a rapid decay to near baseline levels (*F*/*F*_0_=1). Yoda1 induced-[Ca^2+^]_i_ increases were significantly and reversibly inhibited by extracellular 1μM Gd^3+^, a non-selective Piezo1 channel inhibitor to 1.36±0.17 (*N*=6; Figures 3A,B) and 10μM Dooku1, and a pharmacological and selective Piezo1 channel inhibitor to 1.33±0.18 (*N*=12) in *F*/*F*_0_ units, reversibly (Figures 3C,D).

### Pharmacological Piezo1 Channel Inhibitors Suppressed Direct Mechanical Stimulation-Induced [Ca^2+^]_i_ Increases in Human Odontoblasts

**Figure 4:**
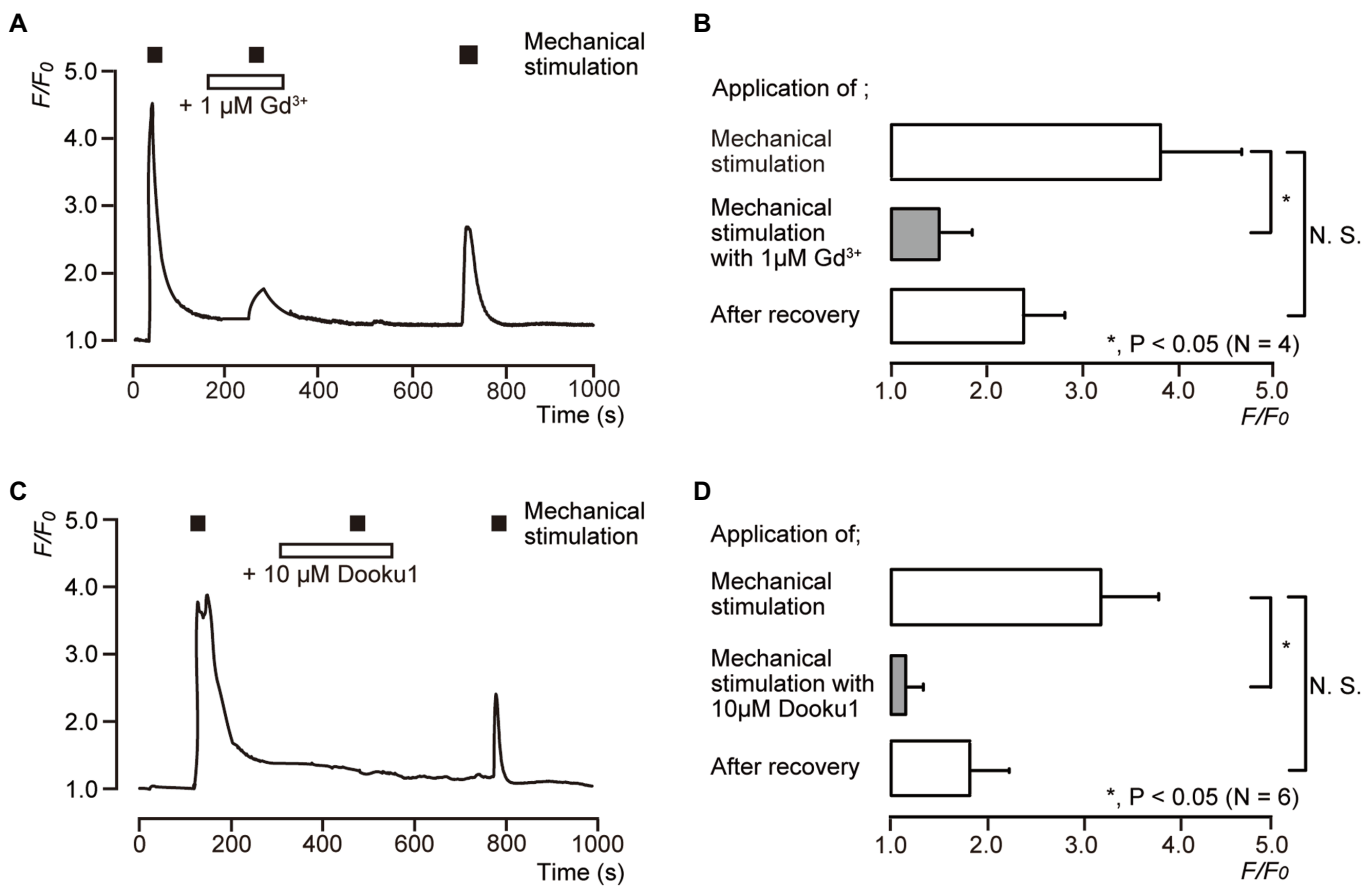
Direct mechanical stimulation-evoked [Ca^2+^]_i_ increases mediated by a Piezo1 channel. **(A,C)** Representative traces of transient increases in [Ca^2+^]_i_ during mechanical stimulations induced by vertical micropipette displacement downward by 8.0μm (black boxes at the top in **A,C**) in standard extracellular solution with (white boxes at the top) or without 1μM Gd^3+^ **(A)** or 10μM Dooku1 **(C)**. **(B,D)** Bar graphs of the values of [Ca^2+^]_i_ increases induced by mechanical stimulation (to 8.0μm) without (open columns) or with (gray columns) pharmacological inhibitors, 1μM Gd^3+^ **(B)**, or 10μM Dooku1 **(D)**. Each of the most lower open columns indicates the *F*/*F*_0_ values after recovery. The resting value is shown as *F*/*F*_0_=1.0. Each bar indicates the mean±SD of four **(B)** and six **(D)** cells tested. Asterisks denote statistically significant differences between columns (shown by solid lines): ^*^p<0.05; N.S., not significant.

We examined the effects of 1μM Gd^3+^ (Figures 4A,B) and 10μM Dooku1 (Figures 4C,D) on the direct mechanical stimulation-induced [Ca^2+^]_i_ increase in single human odontoblasts. The [Ca^2+^]_i_ increases induced by mechanical stimulation [3.81±0.84 in *F*/*F*_0_ units (*N*=4) in Figures 4A,B and 3.16±0.59 in *F*/*F*_0_ units (*N*=6) in Figures 4C,D as the control, respectively] were reversibly and significantly inhibited by the application of 1μM Gd^3+^ to 1.47±0.31 (*N*=4; Figures 4A,B) and 10μM Dooku1 to 1.15±0.15 (*N*=6) in *F*/*F*_0_ units (Figures 4C,D). The [Ca^2+^]_i_ induced by mechanical stimulation was recovered to 2.38±0.43 (*N*=4) after Gd^3+^ and to 1.81±0.41 (*N*=6) after Dooku1 application (Figure 4).

### Piezo1 Channel Knockdown by Short Hairpin RNA Reduced the Functional Expression of Piezo1 but Not Piezo2 Channels

**Figure 5:**
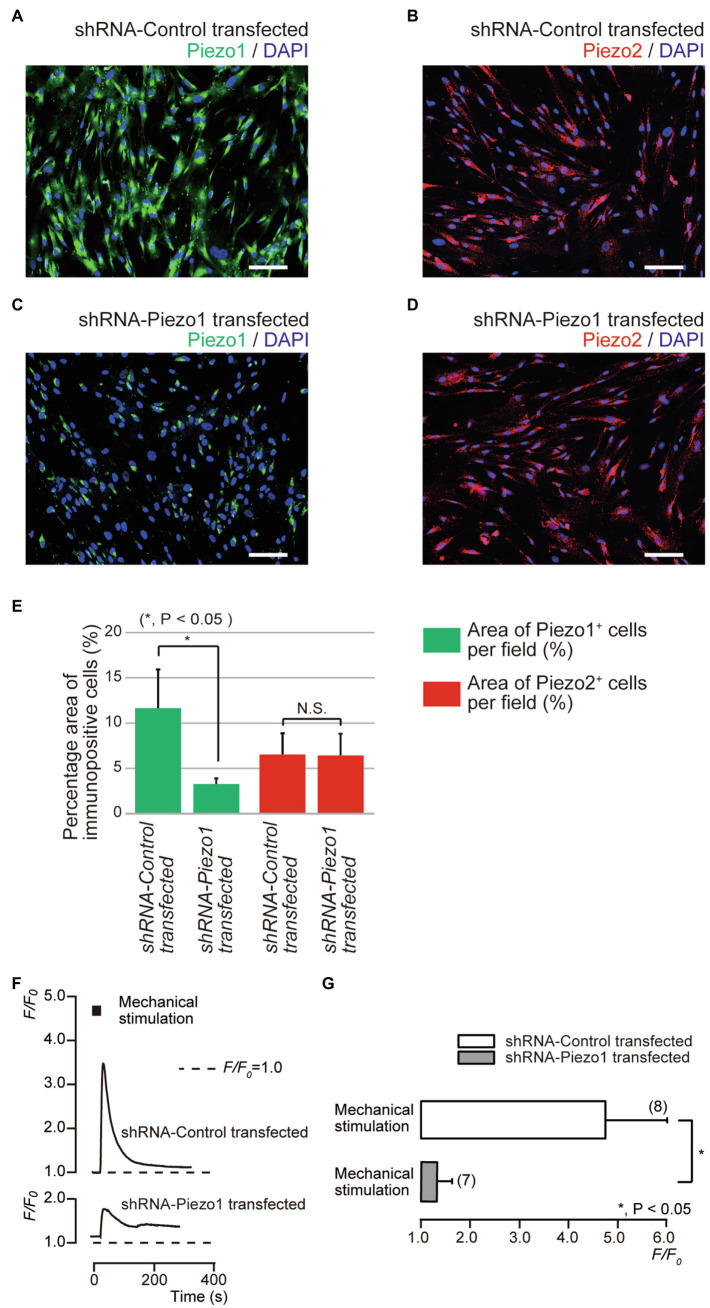
Piezo1 channel knockdown by gene silencing with short hairpin RNA (shRNA) reduced the functional expression of Piezo1 not Piezo2 channels. **(A–D)** Immunoreactivity to the Piezo1 channels (green in **A,C**) or the Piezo2 channels (red in **B,D**) in human odontoblasts transfected by shRNA including a vector specific for human Piezo1 **(C,D)**, or including an empty vector control **(A,B)**. Nuclei are shown in blue. Scale bar: 100μm. No fluorescence was detected in the negative controls (not shown). **(E)** Bar graph showing the percentage area of the Piezo1 channel-immunopositive cells (%; green columns), and those of Piezo2 channel-immunopositive cells (%; red columns) in immunofluorescence analysis from human odontoblasts transfected by shRNA including a vector specific for human Piezo1 (second left and most right columns), or including an empty vector control (most left and third left). Each bar indicates the mean±SD of five experiments. **(F)** Representative traces of transient increases in [Ca^2+^]_i_ during mechanical stimulations induced by vertical micropipette displacement downward by 8.0μm (black boxes at the top) in standard extracellular solution in cells transfected by shRNA including a vector specific for human Piezo1 (shRNA-Piezo1 transfected), or including an empty vector control (shRNA-Control transfected). **(G)** Bar graph of the values of [Ca^2+^]_i_ increases induced by mechanical stimulation (8.0μm) in cells transfected by shRNA including a vector specific for human Piezo1 (gray column), or including an empty vector control (open column). The resting value is shown as *F*/*F*_0_=1.0. The numbers in parentheses indicate the number of cells tested. Asterisks denote statistically significant differences between columns (shown by solid line in G): ^*^p<0.05; N.S., not significant.

We further investigated mechanical stimulation-induced [Ca^2+^]_i_ increases in human odontoblasts transfected with shRNA with a vector specific for human Piezo1, and an empty vector control. In the immunofluorescence analysis, we observed immunoreactivity not only of the Piezo1 channels (green, Figures 5A,E), but also of the Piezo2 channels (red, Figures 5B,E) in the cells transfected with an empty vector control. In the cells transfected by shRNA with a vector specific for human Piezo1 (shRNA-Piezo1 transfected cells), we observed a significant reduction in the percentage area of Piezo1-immunopositive cells (3.14±0.71%; *N*=5; Figures 5C,E) compared to that in the cells transfected by shRNA with an empty vector control (as shRNA-Control transfected cells; 11.5±4.25%; *N*=5; Figures 5A,E). However, we did not observe any significant changes in the area of Piezo2-immunopositive cells in both cells: 6.38±2.44% (*N*=5, Figures 5B,E) in the shRNA-Control transfected cells and 6.29±2.49% (*N*=5, Figures 5D,E) in the shRNA-Piezo1 transfected cells. In addition, in the presence of extracellular Ca^2+^, we observed significant changes in *F*/*F*_0_ units by mechanical stimulation-induced [Ca^2+^]_i_ increases in cells between the shRNA-Piezo1 transfected (1.34±0.29; *N*=7) and shRNA-Control transfected cells (4.74±1.25; *N*=6; Figures 5F,G).

### Activation of Piezo1 Channel by the Pharmacological Modifiers Reduced Mineralization

**Figure 6:**
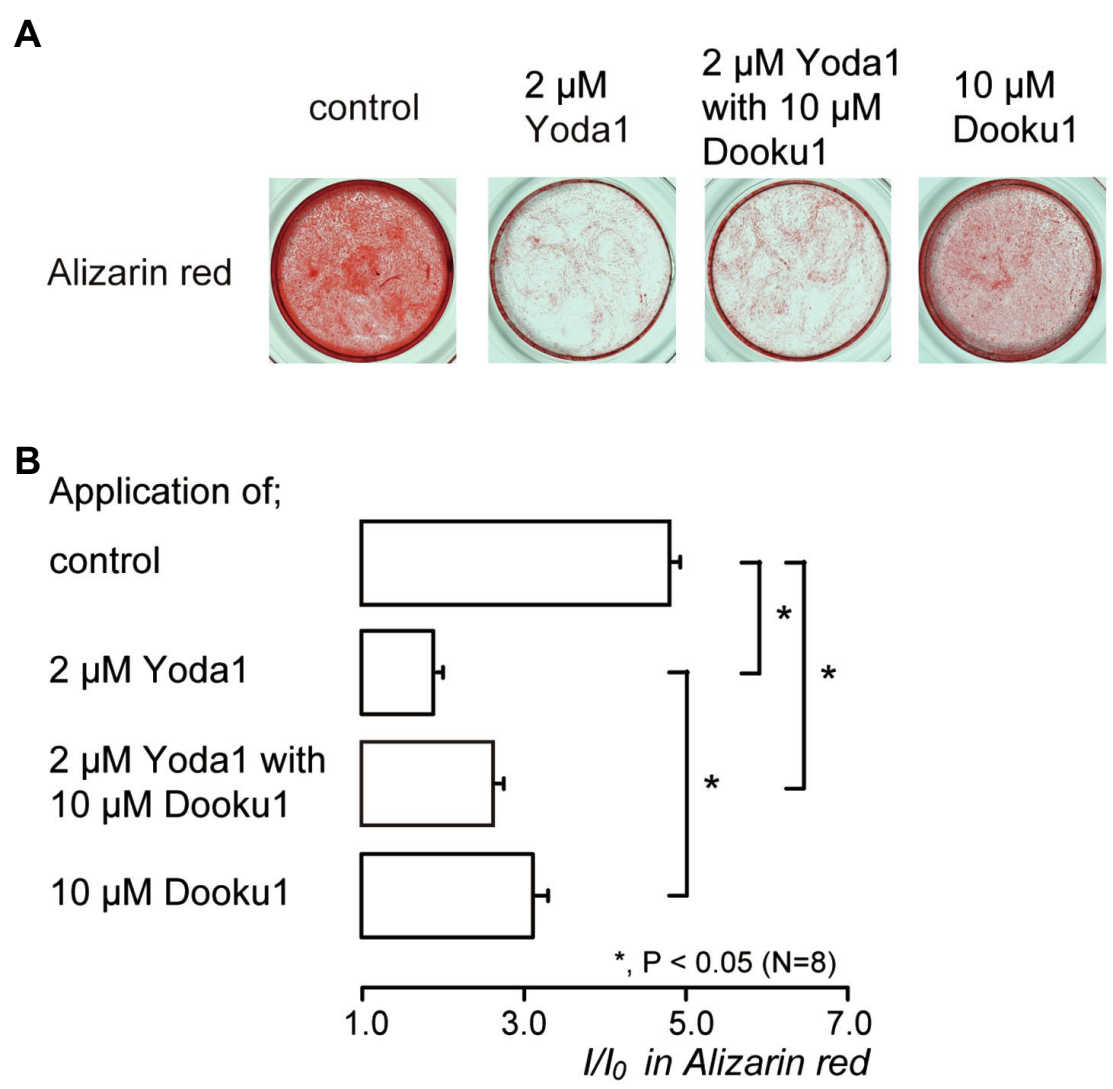
Piezo1 channel activation decreases the mineralization levels. **(A)** Human odontoblasts were cultured for 28days in mineralization medium with 2μM Yoda1 (second left), 2μM Yoda1 and 10μM Dooku1 (third left), 10μM Dooku1 (right most), or without these channel modifiers (left most) at pH 7.4, and stained by Alizarin red (red, calcium deposition). **(B)** Mineralization levels without (control; upper most column) or with 2μM Yoda1 (second column), 2μM Yoda1 and 10μM Dooku1 (third column), and 10μM Dooku1 (lower column), assessed by Alizarin red staining. The mineralization levels were 4.80±0.13 *I*/*I*_0_ in the absence of Piezo1 channel modifiers (as controls), while those 1.96±0.04 *I*/*I*_0_ with 2μM Yoda1, 2.63±0.12 *I*/*I*_0_ with 2μM Yoda1 and 10μM Dooku1, and 3.11±0.18 *I*/*I*_0_ with 10μM Dooku1. Each column denotes the mean±SE from eight experiments. Statistically significant differences between columns (shown by solid lines) are indicated by asterisks. ^*^p<0.05.

We investigated the effects of Piezo1 channel activity on mineralization induced by human odontoblasts. Alizarin red staining (Figure 6A) was determined to be indicative of the mineralization levels based on the staining intensity (see Materials and Methods) represented as *I*/*I*_0_ units; the intensities (*I*) of the stains were normalized to the mean intensities of adjacent areas without cells (*I*_0_; Figure 6B). The application of 2μM Yoda1, or 2μM Yoda1 and 10μM Dooku1, significantly reduced their mineralization levels compared to the level without these pharmacological Piezo1 channel modifiers. In addition, the level in the presence of 2μM Yoda1 was significantly lower than that in the presence of 10μM Dooku1.

### Piezo1 Channel Knockdown by shRNA Enhanced Mineralization

**Figure 7:**
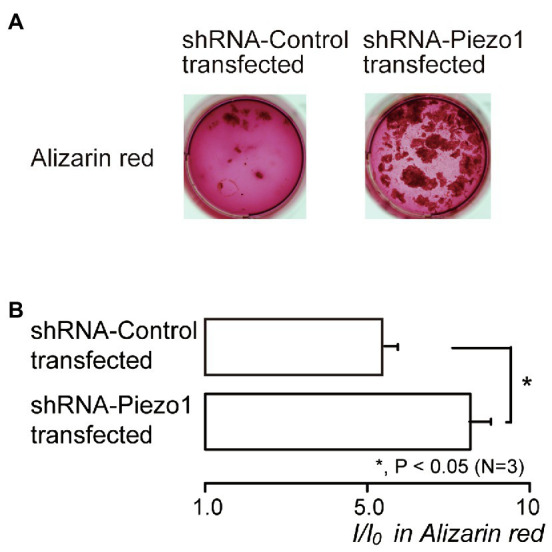
Piezo1 channel knockdown by shRNA enhances the mineralization levels. **(A)** Human odontoblasts transfected by shRNA including a vector specific for human Piezo1 (right; shRNA-Piezo1 transfected) or including an empty vector control (left; shRNA-Control transfected) were cultured for 28days in mineralization medium, and subjected to the Alizarin red staining (red indicative of calcium deposition). **(B)** The mineralization levels in the cells of shRNA-Control transfected (upper column) and shRNA-Piezo1 transfected (lower column), assessed by Alizarin red staining. The mineralization levels were 5.95±0.36 *I*/*I*_0_ in the shRNA-Control transfected cells, and 8.30±0.59 *I*/*I*_0_ in the shRNA-Piezo1 transfected cells. Each column indicates the mean±SE from three experiments. Statistically significant differences between columns (solid line) are indicated by asterisks. ^*^p<0.05.

We also examined the contribution of Piezo1 channel activity to mineralization efficiency. The mineralization level represented by Alizarin red staining (Figure 7A) in the shRNA-Piezo1 transfected cells was significantly higher than that in the shRNA-Control transfected cells (Figure 7B).

## Discussion

The results showed that human odontoblasts, which showed DMP-1-, nestin-, and DSPP-immunoreactivity, functionally express Piezo1 channels activated by Yoda1 pharmacologically and by direct mechanical stimulation. Both Yoda1 induced- and direct mechanical stimulation-induced-[Ca^2+^]_i_ increases were sensitive to extracellular Gd^3+^ and Dooku1, which are pharmacological non-selective and selective Piezo1 channel inhibitors, respectively. In human odontoblasts, mechanical stimulation-induced-[Ca^2+^]_i_ increases also mediated intercellular signal networks among odontoblasts, as previously reported in rat studies (Sato et al., 2015; Shibukawa et al., 2015). These mechanical stimulation-induced [Ca^2+^]_i_ responses were almost completely abolished in cells when Piezo1 channels were knocked down by shRNA. In addition, Alizarin red staining indicated that pharmacological Piezo1 channel activation by Yoda1 significantly suppressed mineralization, and the shRNA-mediated knockdown of Piezo1 significantly enhanced mineralization.

We previously found that odontoblasts can detect mechanical stimuli as cellular deformation originating from dentinal fluid movement by dentin stimuli and act as sensory receptor cells to generate dentinal sensitivity. The mechanism underlying the generation of dentinal sensitivity has been termed the odontoblast hydrodynamic receptor model (Shibukawa et al., 2015). The stimuli to the dentin surface transformed into dentinal fluid movements and induced cell membrane deformation in odontoblasts. Cell deformation activates mechanosensitive transient receptor potential (TRP) channels, such as TRP vanilloid subfamily member 1 (TRPV1), TRPV2, TRPV4, and TRP ankyrin 1 (TRPA1) channels, increasing [Ca^2+^]_i_ through Ca^2+^ influx (Tsumura et al., 2012, 2013; Sato et al., 2013, 2018; Shibukawa et al., 2015). The [Ca^2+^]_i_ increase established intercellular odontoblast-odontoblast and odontoblast-neuron signal communication mediated by extracellular signaling molecules, ATP, and glutamate, which are released from mechanically stimulated odontoblasts (Sato et al., 2015; Shibukawa et al., 2015; Nishiyama et al., 2016). The released ATP (from pannexin-1 channels in odontoblasts triggered by the [Ca^2+^]_i_ increase) activates ionotropic ATP receptor subtype 3 (P2X_3_ receptors) in the intradental Aδ neurons. The activation of P2X_3_ receptors induces an action potential in pulpal Aδ neurons, indicative of the generation of dentinal sensitivity (Sato et al., 2018). Additionally, in line with the present results, the application of selective Piezo1 channel inhibitors, GsMTx4, also inhibited synaptic-like responses in TG neurons evoked by the direct mechanical stimulation of odontoblasts. Together with the present and previous results, Piezo1 channels in odontoblasts are involved in detecting cell membrane deformation due to dentinal fluid movements by the stimuli applied to the dentin surface to generate dentinal sensitivity, and in mediating inter-odontoblast signal networks. Indeed, the inhibition of Piezo1 channels by the systemic administration of GsMTx4 and the somatic elimination of odontoblasts in the dentin-pulp border (using Cre/LoxP-based technology; Zhao et al., 2021) almost completely abolished nociceptive behaviors by cold stimuli to the exposed dentin surface in rats/transgenic mice showing dentinal sensitivity (personal communication from YS).

In line with our results, it has been described that Piezo1 channels are expressed not only in multipotent stem cells from human exfoliated deciduous teeth (SHED), but also in odontoblast processes (Miyazaki et al., 2019). In addition to the mechanosensory transduction roles of odontoblasts, Piezo1 channels also mediate hydrostatic pressure-induced differentiation of odontoblasts from SHED (Miyazaki et al., 2019). In the Alizarin red staining of the present study, however, the pharmacological activation of the Piezo1 channel significantly suppressed mineralization efficiency. In addition, the intensity of Alizarin red staining of shRNA-Piezo1 transfected cells was significantly higher than that in shRNA-Control transfected cells. These results indicate that Piezo1 channels in odontoblasts may negatively regulate not only reactionary dentinogenesis induced by cell membrane deformation due to dentinal fluid movements by the stimuli applied to the exposed dentin surface, but also physiological/developmental dentinogenesis. In contrast, the activation of mechanosensitive- and high-pH sensitive-TRPA1 channels in odontoblasts positively modulates mineralization in physiological, developmental, and pathological conditions [e.g., Ca(OH)_2_ or mineral trioxide aggregate (MTA) application on dentin; Kimura et al., 2016]. Although further studies are needed, these convergent results suggest that, among odontoblast-expressing mechanosensitive cation channels, including TRPV1, TRPV2, TRPV4, TRPA1, and Piezo1 channels, their functional roles in sensory transduction and dentin formation are different.

Recent evidence have indicated that mouse odontoblasts also express Piezo2 channels (Khatibi Shahidi et al., 2015; Krivanek et al., 2020). In line with these results, we successfully observed Piezo2-immunoreactivity in both shRNA-Piezo1 transfected and shRNA-Control transfected cells. Thus, we could not exclude the possibility that Piezo2 channels are also involved in mechanosensory transduction in odontoblasts. However, further study will be needed to examine how Piezo2 channels contribute to the sensory transduction sequences, and what differences exist between the roles of Piezo1 and Piezo2 channels in cellular function. In addition, in endothelial cells, it has been demonstrated that Piezo1 channels regulate TRPV4 channel openings. In the present study, the pharmacological Piezo1 inhibitors Gd^3+^ (1μM) and Dooku1 (10μM) almost completely suppressed direct mechanical stimulation-induced [Ca^2+^]_i_ increases in single human odontoblasts. We also only observed small residual responses in mechanical stimulation-induced [Ca^2+^]_i_ increases in shRNA-Piezo1 transfected cells compared to those in the shRNA-Control transfected cells. Therefore, together with the present results, Piezo1 channels may act upstream of mechanosensitive cation channels, including TRPV1, TRPV2, TRPV4, and TRPA1 channels, as well as Piezo2 channels, in odontoblasts.

In conclusion, we demonstrated that human odontoblasts functionally express Piezo1 channels, and mechanical stimulation-induced-[Ca^2+^]_i_ increases mediate the intercellular signaling networks among them. Piezo1 channels predominantly contribute to the detection of cellular deformation, such as mechanical stimulation, induced by dentinal fluid movements in dentinal tubules, during various stimuli applied to the exposed human dentin surface, such as enamel lesions including dental caries and/or acid erosion. However, Piezo1 channel activity negatively regulates not only reactionary dentinogenesis, but also physiological/developmental dentinogenesis. This suggests that Piezo1 channel activity may physiologically prevent the lifelong loss of volume in the pulp chamber by excessive dentin formation induced by mechanical stimuli applied on the teeth, such as mastication.

## Data Availability Statement

The original contributions presented in the study are included in the article/supplementary material; further inquiries can be directed to the corresponding author.

## Author Contributions

MM, MK, TI, and YS conceived and designed the experiments. MM, MK, TO, TN, SO, MA, SN, and TA performed the experiments. MM, MK, TO, TN, SO, MA, SN, TA, TI, and YS were responsible for analyzing and interpreting the data and drafting and critically revising the intellectual content of the article. TI and YS were responsible for the final approval of the version to be submitted or published. All authors contributed to the article and approved the submitted version.

## Conflict of Interest

The authors declare that the research was conducted in the absence of any commercial or financial relationships that could be construed as a potential conflict of interest.

## Publisher’s Note

All claims expressed in this article are solely those of the authors and do not necessarily represent those of their affiliated organizations, or those of the publisher, the editors and the reviewers. Any product that may be evaluated in this article, or claim that may be made by its manufacturer, is not guaranteed or endorsed by the publisher.
